# The Use of Long-Acting Injectable Antipsychotics (LAI) in the Serious Mental Illness (SMI) Patients Enrolled in an Assertive Community Treatment (ACT) Program

**DOI:** 10.7759/cureus.14490

**Published:** 2021-04-14

**Authors:** Maria Ruiza Yee, Eduardo Espiridon, Adeolu O Oladunjoye, Udema Millsaps, Anish Vora, Nailah Harvey

**Affiliations:** 1 Psychiatry, Drexel University College of Medicine, Philadelphia, USA; 2 Psychiatry, Philadelphia College of Osteopathic Medicine, Philadelphia, USA; 3 Psychiatry, Reading Hospital Tower Health, West Reading, USA; 4 Psychiatry, Tower Health Medical Group, West Reading, USA; 5 Critical Care Medicine, Boston Children's Hospital, Boston, USA

**Keywords:** lai, act, serious mental illness

## Abstract

Introduction

Patients with serious mental illness (SMI) experience highly intractable symptoms and great levels of dysfunction from their mental illness. Relapse prevention is critical as psychopathology, social and occupational functioning worsen with repeated psychotic episodes. Poor medication adherence is a strong predictor of relapse. Use of long-acting injectable antipsychotics (LAI) is among the most effective treatment specially in the context of non-adherence and yet remains underutilized. This single center retrospective study conducted using the electronic medical record (EMR) of patients enrolled in an Assertive Community Treatment (ACT) program at a community hospital was analyzed as to whether use of LAI among these patients reduce the frequency of emergency room visits and hospitalizations.

Materials and methods

Single center retrospective study using EMR of patients ages 20 and above who were enrolled at the ACT program at a community hospital from December 1, 2008 to December 31, 2018. Variables were collected from the EMR and de-identified into an Excel spreadsheet for data collation. Analysis was performed using SPSS software package.

Results

A total of 179 patients enrolled in the ACT program and their hospitalizations were extracted from the EMR. Seventy-six (42.5%) of these hospitalizations had patients on LAI. The hospitalizations were made up of 53.6% male, 81.9% White/Asian, 18.1% Black; 44.1% ages between 36 and 50 years old, 30.2% ages between 18 and 35 years old, and 25.7% greater than 50 years old. There was no difference in age, sex, race, ethnicity, insurance type and time spent in ACT program between those using LAI and those not on LAI. There was a higher proportion of psychiatric hospitalizations among LAI users compared with the non-LAI user group (57.9% vs 37.4%, p = 0.007). However, the two groups did not differ from one another in terms of psychiatric emergency visits (p = 0.266) or frequency of ACT visits (p = 0.062).

Conclusion

To date, all of the new-generation antipsychotic LAI have demonstrated a statistically and clinically significant decrease of relapse rates over placebo. Despite this, LAIs are not widely prescribed for a variety of reasons, including the reservations of patients, clinicians and payers. It would seem, though, that our patient population at the ACT program do not seem to benefit from use of LAI in relapse prevention. These results are counterintuitive in that one would expect that patients with serious mental illness would benefit from use of LAI. Perhaps, individuals with SMI are a different subset of population and they do not respond as well to LAI.

## Introduction

Serious mental illness (SMI), as defined by the National Institute of Mental Health (NIMH) is a mental, behavioral or emotional disorder resulting in serious functional impairment which substantially interferes with or limits one or more major life activities [1]. It is a more severe and smaller subset of mental illness. According to the NIMH, as of 2019, there are approximately 13.1 million or 5.2% of American adults age 18 or over, who have SMI. Young adults, age 18 to 25, have the highest prevalence. Females, compared to males, are at increased risk of suffering from SMI. The economic burden of these individuals is substantial: not just the cost of direct health care but also the loss of income due to unemployment, expenses for social supports and a range of indirect costs due to chronic disability [2]. Hence, it is important to prevent relapses and ensure clinical stability in this patient population.

Patients with SMI involved in an Assertive Community Treatment (ACT) program are individuals who experience the most intractable symptoms and the greatest level of dysfunction from their mental illness. These individuals typically are institutionalized in a long-term facility such as the state hospital. Patients who suffer from SMI do not respond to standard outpatient psychiatric care. There is ample evidence that ACT is an effective [3-5], and cost-effective treatment [6-8] in this group of individuals, especially in those with extensive prior hospital use. ACT program not only results in reduction of psychiatric hospitalization but also, a more stable housing.

ACT originated over 30 years ago when a group of mental health professionals at the Mendota Mental Health Institute in Wisconsin [4] recognized the high rate of recidivism in individuals with SMI who were discharged in stable condition. They determined what could be done and designed this service delivery model. A team of 10 to 12 professionals from the fields of psychiatry, psychology, nursing, social work, vocational rehabilitation, drug and alcohol addiction, and peer support, is responsible for providing the services needed by these patients. The team ensures services are available 24 hours a day, seven days a week. Unlike standard psychiatric care where patients are taught skills that are expected to be generalized to real life situations, services are provided in the context and setting in which problems arise and support or skills are needed.

Relapse prevention is critical as psychopathology, social and occupational functioning worsen with repeated psychotic episodes. Relapse also leads to higher healthcare costs. Pharmacotherapy is often limited due to high rates of non-adherence, especially when oral antipsychotic medications are used. Poor medication adherence is also a strong predictor of relapse [9,10]. Long-acting injectable antipsychotics (LAI) is among the most effective treatment, especially in the context of non-adherence, and yet remains underutilized [11,12].

The purpose of this study is to ascertain whether the addition of LAI to ACT treatment of SMI patients led to a decrease in the frequency of hospitalizations and increase in clinical stability.

## Materials and methods

This was a single center retrospective study conducted using the electronic medical record (EMR). The cohort patient population studied included patients ages 20 and above who were enrolled at an ACT program at a community hospital from December 1, 2008 to December 31, 2018.

Abstracted data from EMR included diagnoses using ICD-10 codes. They include Manic episode F 30.x; Bipolar disorder F 31.x; Schizophrenia F 20.x; Schizoaffective disorder F 25.x. Also, for years before 2016, abstracted data included diagnoses using ICD-9 diagnostic codes. They include Schizophrenic disorders 295.x; Episodic mood disorders 296.x, and Delusional disorders 297.x.

Data on medications given during the study period to these patients were also extracted. These include LAI such as fluphenazine, haloperidol, risperidone, aripiprazole, and paliperidone. Oral antipsychotic medications, both typical and atypical, and other medications given to manage the patient’s condition, and the dosage and dates when they were given, were also recorded.

Information on a total of 189 patients were extracted from the EMR which included patients with at least one hospitalization. There were five patients who dropped out of the ACT program and five patients with incomplete/inappropriate data information. These patients were excluded from the final analysis, leaving the study population to 179 patients. Eight patients had two separate encounters in the ACT program while 163 patients had only one encounter in the ACT program. Patient hospitalizations were divided into two arms, i.e., those who received LAI and those who did not receive LAI.

Possible limitation of this method is misclassification of the patient’s diagnostic code information during their index visit at ACT enrollment. However, statistical errors were minimized by excluding patients who have vague diagnosis during data collection. This means of collection of data is the best way to assess the population of psychiatric patients receiving this unique type of care.

Variables were collected from the EMR and de-identified into an Excel spreadsheet for data collation. Data were stored in a secure password protected server. Analysis was performed using SPSS software (IBM Corp., Armonk, NY) package.

Statistical analysis

The statistical analysis described the prevalence of LAI among SMI patients enrolled in the ACT program. Summary statistics are presented using proportions, mean, and standard deviation. Mean number of hospitalizations, emergency visits and number of planned outpatient/ACT visits in the two groups (LAI vs non-LAI treatment) were compared.

Multivariate linear regression analysis was used to adjust for other independent co-variables. Kaplan-Meier analysis was used to compare time from ACT enrollment to first psychiatric hospitalization. The level of significance was set at p < 0.05. All statistical analyses were performed using SPSS software package.

## Results

A total of 179 hospitalizations were analyzed. In 76 (42.5%) hospitalizations, LAI was used during the study. The hospitalizations were made up of 53.6% male, 46.4% female, 81.9% White/Asian, 18.1% Black, 44.1% 36 to 50 years old, 30.2% 18 to 35 years old and 25.7% greater than 50 years old. The overall mean age was 42.5 $\pm$11.8 years (Table 1). Table 1 describes the demographic and clinical characteristics of patients with SMI enrolled at the ACT program who were placed on LAI. Insurance coverage varied with Medicaid insurance (56.5%) being the predominant insurance coverage, followed by Medicare insurance (36.2%), and private insurance or workman's compensation (7.3%). Most of the patients were unemployed or retired (95.9%). The most common diagnosis is schizoaffective disorder (44.0%), followed by schizophrenia (35.4%), bipolar disorder (16.0%) and major depressive disorder (4.6%). Co-morbid substance abuse was significant with alcohol being the most commonly abused at 54.2%, marijuana at 44.1% and cocaine at 29.6%. The overall substance use disorder in the cohort was 71.5%. Most of the patients were enrolled in the ACT program, an average of one to five years (46.7%).

**Table 1:** Baseline and clinical characteristics of patients with serious mental illness (SMI) enrolled at the Assertive Community Treatment (ACT) program with use of Long-Acting Injectable Antipsychotic (LAI) and without use of Long-Acting Injectable Antipsychotic (LAI).

Name	All SMI (n = 179)	LAI Use (n = 76)	No LAI Use (n = 103)	P Value
Mean Age (±SE)	42.5±11.8	41.9±12.1	43.0±11.6	
Age, years				
18-35	54 (30.2%)	25 (32.9%)	29 (28.2%)	
36-50	79 (44.1%)	33 (43.4%)	46 (44.7%)	
>50	46 (25.7%)	18 (23.7%)	28 (27.2%)	0.760
Sex				
Female	83 (46.4%)	31 (40.8%)	52 (50.5%)	
Male	96 (53.6%)	45 (59.2%)	51 (49.5%)	0.199
Race				
White/Asian	145 (81.9%)	62 (83.8%)	83 (80.6%)	
Black	32 (18.1%)	12 (16.2%)	20 (19.4%)	0.585
Ethnicity				
Hispanic	44 (24.6%)	26 (34.2%)	18 (17.5%)	
Non-Hispanic	135 (75.4%)	50 (65.8%)	85 (82.5%)	0.010
Insurance				
Medicaid	100 (56.5%)	44 (58.7%)	56 (54.9%)	
Medicare	64 (36.2%)	27 (36.0%)	37 (36.3%)	
Private/ Worker’s compensation	13 (7.3%)	4 (5.3%)	9 (8.8%)	0.662
Employment				
Unemployed/Retired	162 (95.9%)	72 (97.3%)	90 (94.7%)	
Employed/Self-Employed	7 (4.1%)	2 (2.7%)	5 (5.3%)	0.469
Diagnosis				
Bipolar Disorders	28 (16.0%)	9 (12.2%)	19 (18.8%)	
Major Depressive Disorder	8 (4.6%)	0 (0.0%)	8 (7.9%)	
Schizoaffective Disorders	77 (44.0%)	32 (43.2%)	45 (44.6%)	
Schizophrenia	62 (35.4%)	33 (44.6%)	29 (28.7%)	0.005
Substance use disorder				
No	51 (28.5%)	20 (26.3%)	31 (30.1%)	
Yes	128 (71.5%)	56 (43.8%)	72 (69.9%)	0.580
Marijuana	79 (44.1%)	40 (52.6%)	39 (37.9%)	0.049
Cocaine	53 (29.6%)	20 (26.3%)	33 (32.0%)	0.407
Alcohol	97 (54.2%)	41 (53.9%)	56 (54.4%)	0.955
Smoking				
No	44 (24.6%)	17 (22.4%)	27 (26.2%)	
Yes	126 (70.4%)	57 (75.0%)	69 (67.0%)	0.338
Days in ACT program				
< 1 year	58 (42.3%)	16 (30.4%)	42 (46.2%)	
1-5 years	64 (46.7%)	23 (43.5%)	41 (45.1%)	
> 5 years	15 (10.9%)	7 (26.1%)	8 (8.8%)	0.325
Number of Psychiatric hospitalizations				
0 No visit	94 (53.7%)	32 (42.1%)	62 (62.6%)	
≥ 1 visit	81 (46.3%)	44 (57.9%)	37 (37.4%)	0.007
Number of Psychiatric Emergency visits				
0 No visit	109 (64.1%)	44 (59.5%)	65 (67.7%)	
≥ 1 visit	61 (35.9%)	30 (40.5%)	31 (32.3%)	0.266
Planned ACT outpatient visits				
<100 visits	36 (20.6%)	10 (13.3%)	26 (26.0%)	
100-500 visits	82 (46.9%)	35 (46.7%)	47 (47.0%)	
>500 visits	57 (32.6%)	30 (40.0%)	27 (27.0%)	0.062

There was no difference in age, sex, race, ethnicity, insurance type and time spent in ACT program between those using LAI and those not on LAI. There was a higher proportion of psychiatric hospitalizations among LAI users compared with the non-LAI user group (57.9% vs 37.4%, p = 0.007). However, the two groups did not differ from one another in terms of psychiatric emergency visits (p = 0.266). Among the LAI group, the LAI used were as follows: risperidone (17.3%), paliperidone (14.0%), haloperidol decanoate (12.3%), aripiprazole (8.9%), and fluphenazine (3.9%) (Table 2).

**Table 2:** Types of long-acting injectable antipsychotic (LAI) used by serious mental illness (SMI) patients in the Assertive Community Treatment (ACT) program.

Type of LAI used	Proportion hospitalizations using Long acting injectable
Paliperidone	25 (14.0%)
Aripiprazole (Abilify Maintena)	16 (8.9%)
Aripiprazole lauroxil (Aristada)	3 (1.7%)
Risperidone	31 (17.3%)
Fluphenazine (Prolixin)	7 (3.9%)
Haloperidol (Haldol) decanoate	22 (12.3%)

Association of psychiatric hospitalizations with LAI use

With univariate analysis, LAI use was associated with psychiatric hospitalization (OR 2.30, 95% 1.25-4.24, p = 0.007). Males were less likely to have psychiatric hospitalization (OR 0.47, CI 0.26-0.87, p = 0.015). Those with substance use disorders were less likely to have psychiatric hospitalizations compared to those who did not have substance use disorder (OR 0.46, 95% CI 0.24-0.90, p = 0.023) (Table 3).

After adjusting for other variables, LAI use was still associated with psychiatric hospitalization (OR 3.08, 95% 1.36-6.97, p = 0.007). Sex also remained an independent risk factor for psychiatric hospitalization with males being less likely to be hospitalized (OR 0.24, 95% CI 0.11-0.52, p < 0.001) (Table 3).

**Table 3:** Association of psychiatric hospitalizations with long-acting injectable antipsychotic (LAI) use in serious mental illness (SMI) at the Assertive Community Treatment (ACT) Program.

Name	Univariate analysis (crude OR)	P Value	Multivariate analysis (adjusted OR) ‡	P Value
LAI use	2.30 (1.25-4.24)	0.007*	3.08 (1.36-6.97)	0.007
Age, years				
18-35	Ref			
36-50	0.96 (0.48-1.93)	0.909		
>50	0.93 (0.42-2.08)	0.866		
Sex				
Female	Ref			
Male	0.47 (0.26-0.87)	0.015*	0.24 (0.11-0.52)	<0.0001
Race				
White/Asian	Ref			
Black	0.81 (0.37-1.77)	0.596		
Ethnicity				
Hispanic	Ref			
Non-Hispanic	0.64 (0.32-1.28)	0.206		
Insurance				
Medicaid	Ref			
Medicare	0.99 (0.52-1.86)	0.964		
Private/ Worker’s compensation	0.99 (0.31-3.16)	0.987		
Employment				
Unemployed/Retired	Ref			
Employed/Self-Employed	0.43 (0.08-2.29)	0.324		
Diagnosis				
Bipolar Disorders	Ref			
Major Depressive Disorder	0.60 (0.12-3.01)	0.534		
Schizoaffective Disorders	0.90 (0.38-2.14)	0.807		
Schizophrenia	0.85 (0.35-2.08)	0.719		
Substance use disorder				
No	Ref			
Yes	0.46 (0.24-0.90)	0.023*	0.78 (0.34-1.78)	0.547
Marijuana	0.82 (0.45-1.50)	0.521		
Cocaine	0.64 (0.33-1.23)	0.179		
Alcohol	0.63 (0.35-1.15)	0.131		
Smoking				
No	Ref			
Yes	0.97 (0.48-1.94)	0.922		
Years in ACT program				
< 1 year	Ref			
1-5 years	1.90 (0.91-3.96)	0.088	1.72 (0.77-3.82)	0.184
> 5 years	2.50 (0.78-8.02)	0.124		
Planned ACT outpatient visits				
<100 visits	Ref			
100-500 visits	1.63 (0.71-3.74)	0.253		
>500 visits	1.81 (0.75-4.37)	0.185		

**Figure 1:**
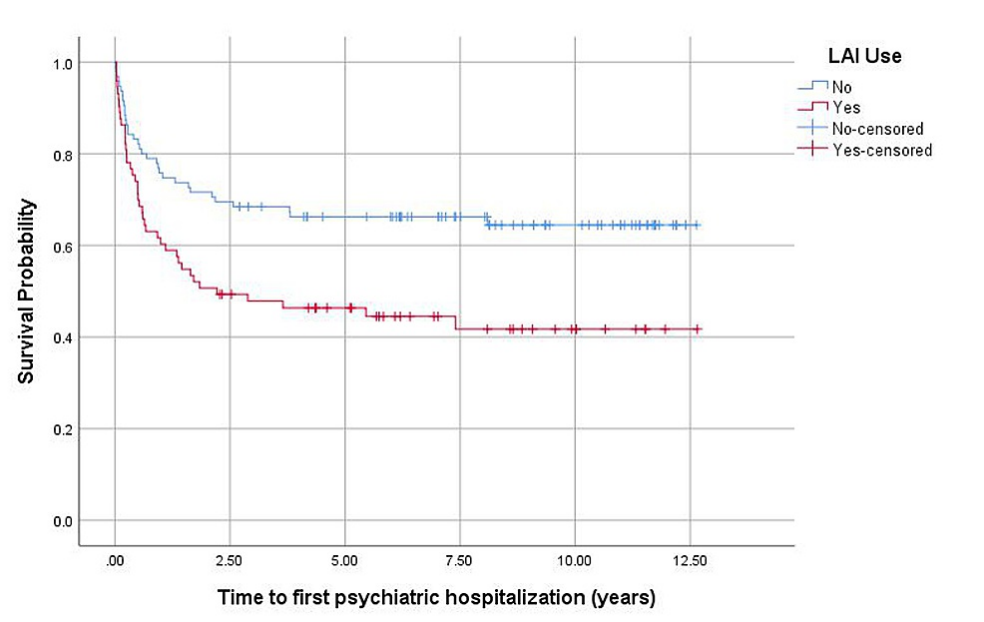
Plot comparing probability of psychiatric hospitalization by long-acting injectable antipsychotic (LAI) use. Kaplan-Meier curve analysis.

The mean time to first psychiatric hospitalization was 5.99 years for those on LAI while it was 8.58 years for those non-LAI group (p = 0.005). At 2.22 years, 50% of those on LAI and 30% of those not on LAI had experienced a psychiatric hospitalization. The probability of psychiatric hospitalization at one and five years were higher in the LAI group (40% and 54%, respectively) than the non-LAI group (24% and 34%, respectively) (p = 0.005) (Figure 1).

## Discussion

Health care cost is one of the most confounding challenges of public policy in the United States. In 2006, health care costs constituted 16% of the gross national product, of which 6.2% is from mental health [2]. With the estimated prevalence of SMI at 13.1 million or 5.2% of American adults age 18 or over, mental health care cost for this patient population is significant [1]. Though SMI is a smaller subset of mental illness, it is more severe. Therefore, healthcare cost in individuals with SMI is substantial. Health care costs include not just the cost of direct health care but also the loss of income due to unemployment, expenses for social supports and a range of indirect costs due to chronic disability [2]. Relapse prevention is critical as psychopathology, social and occupational functioning worsen with repeated psychotic episodes. Relapse also leads to higher healthcare costs. Hence, it is important to prevent relapses and ensure clinical stability in this patient population.

Individuals with SMI do not respond to standard psychiatric services. Over the course of the last 30 years, ACT has proven to be not only an effective [3-5] but also a cost-effective treatment [6-8] in this group of individuals, especially in those with extensive prior hospital use. ACT program not only results in reduction of psychiatric hospitalization but also, a more stable housing.

Pharmacotherapy is often limited due to high rates of non-adherence, especially when oral antipsychotic medications are used. Poor medication adherence is also a strong predictor of relapse [9,10]. LAI is among the most effective treatment, especially in the context of non-adherence, and yet remains underutilized [11,12]. It made sense to explore the use of LAI in this patient population already receiving ACT. Use of LAI would certainly improve adherence, and therefore, prevent relapse.

Surprisingly, the results of our study do not substantiate the efficacy of LAI in this patient population. If anything, it was associated with increased hospitalizations. This is despite the high level of adherence to the LAI that was prescribed. It would seem that use of LAI was not contributory in maintaining clinical stability in this patient population. Perhaps patients with SMI, already being treated at ACT program, constitute a very severe patient population and that in itself, is a predictor of poor outcome.

## Conclusions

To date, all of the new-generation antipsychotic LAI have demonstrated a statistically and clinically significant decrease of relapse rates over placebo. Despite this, LAIs are not widely prescribed for a variety of reasons, including the reservations of patients, clinicians and payers. It would seem, though, that our patient population at the ACT program do not seem to benefit from use of LAI in relapse prevention. These results are counterintuitive in that one would expect that patients with serious mental illness would benefit from use of LAI. Perhaps, individuals with SMI already involved in ACT program are a different subset of population and they do not respond as well to LAI. Also, the outcome of the study may be different if the focus was limited to the relationship to hospitalization, before and after receiving the LAI, to show whether the LAI can prevent relapse and hospitalization.

## References

[1] (2021). National Institute of Mental Health. Mental illness. https://www.nimh.nih.gov.

[2] Insel TR (2008). Assessing the economic costs of serious mental illness. Am J Psychiatry.

[3] Udechuku A, Olver J, Hallam K (2005). Assertive community treatment of the mentally ill: service model and effectiveness. Australas Psychiatry.

[4] Phillips S, Burns B, Edgar E (2001). Moving assertive community treatment into standard practice. Psychiatri Serv.

[5] Burns BJ, Santos AB (1995). Assertive community treatment: an update of randomized trials. Psychiatr Serv.

[6] Bond GR, Drake RE, Mueser KT, Latimer E (2001). Assertive community treatment for people with severe mental illness. Dis Manage Health Outcomes.

[7] Latimer EA (1999). Economic impacts of assertive community treatment: a review of the literature. Can J Psychiatry.

[8] Rosenheck R, Neale M (1998). Intersite variation in the impact of intensive psychiatric community care on hospital use. Am J Orthopsychiatry.

[9] Novick D, Haro JM, Suarez D, Perez V, Dittmann RW, Haddad PM (2010). Predictors and clinical consequences of non-adherence with antipsychotic medication in the outpatient treatment of schizophrenia. Psychiatry Res.

[10] Kane JM, Kishimoto T, Correll CU (2013). Non-adherence to medication in patients with psychotic disorders: epidemiology, contributing factors and management strategies. World Psychiatry.

[11] Miyamoto S, Wolfgang Fleischhacker W (2017). The use of long-acting injectable antipsychotics in schizophrenia. Curr Treat Options Psychiatry.

[12] Heres S (2014). Long-acting injectable antipsychotics: an underutilized treatment option. J Clin Psychiatry.

